# Beyond gait speed: exploring the added value of Inertial Measurement Unit-based measurements of gait in the estimation of the walking ability in daily life

**DOI:** 10.1186/s12883-024-03632-0

**Published:** 2024-04-17

**Authors:** R. A. W. Felius, N. C. Wouda, M. Geerars, S. M. Bruijn, J. H. van Dieën, M. Punt

**Affiliations:** 1 Research group lifestyle and health, Utrecht University of Applied Sciences, Utrecht, The Netherlands; 2 Department of Human Movement Science, Vrije Universiteit Amsterdam, Amsterdam, The Netherlands; 3 Physiotherapy Department Neurology, Axioncontinu, Rehabilitation Center de Parkgraaf, Utrecht, The Netherlands; 4 Center of Excellence for Rehabilitation Medicine, UMC Utrecht Brain Center, University Medical Center Utrecht, Utrecht University and De Hoogstraat Rehabilitation, Utrecht, the Netherlands; 5 Department of neurorehabilitation, De Hoogstraat Rehabilitation, Utrecht, The Netherlands

**Keywords:** Accelerometer, Cerebrovascular accident, Daily life gait characteristics, Functional gait assessment, Gait quality, Inertial measurement units, Stroke recovery, Walking ability

## Abstract

**Background:**

Gait speed is often used to estimate the walking ability in daily life in people after stroke. While measuring gait with inertial measurement units (IMUs) during clinical assessment yields additional information, it remains unclear if this information can improve the estimation of the walking ability in daily life beyond gait speed.

**Objective:**

We evaluated the additive value of IMU-based gait features over a simple gait-speed measurement in the estimation of walking ability in people after stroke.

**Methods:**

Longitudinal data during clinical stroke rehabilitation were collected. The assessment consisted of two parts and was administered every three weeks. In the first part, participants walked for two minutes (2MWT) on a fourteen-meter path with three IMUs attached to low back and feet, from which multiple gait features, including gait speed, were calculated. The dimensionality of the corresponding gait features was reduced with a principal component analysis. In the second part, gait was measured for two consecutive days using one ankle-mounted IMU. Next, three measures of walking ability in daily life were calculated, including the number of steps per day, and the average and maximal gait speed. A gait-speed-only Linear Mixed Model was used to estimate the association between gait speed and each of the three measures of walking ability. Next, the principal components (PC), derived from the 2MWT, were added to the gait-speed-only model to evaluate if they were confounders or effect modifiers.

**Results:**

Eighty-one participants were measured during rehabilitation, resulting in 198 2MWTs and 135 corresponding walking-performance measurements. 106 Gait features were reduced to nine PCs with 85.1% explained variance. The linear mixed models demonstrated that gait speed was weakly associated with the average and maximum gait speed in daily life and moderately associated with the number of steps per day. The PCs did not considerably improve the outcomes in comparison to the gait speed only models.

**Conclusions:**

Gait in people after stroke assessed in a clinical setting with IMUs differs from their walking ability in daily life. More research is needed to determine whether these discrepancies also occur in non-laboratory settings, and to identify additional non-gait factors that influence walking ability in daily life.

**Supplementary Information:**

The online version contains supplementary material available at 10.1186/s12883-024-03632-0.

## Background

One of the main rehabilitation goals for people after stroke is to regain the ability to walk in daily life, i.e. to ambulate independently inside and outside their home [1–3]. During rehabilitation, this ability is often estimated with a measurement of gait speed, for instance a two-minute walk test (2MWT) [4–8]. Evidence suggests there is a strong association between gait speed and the walking ability; a higher gait speed is linked to increased community ambulation [4–6].

Nowadays, it has become feasible to collect additional information about gait, e.g. the stability, regularity and symmetry of the gait pattern, during a 2MWT, for instance with inertial measurement units (IMUs). Prior research has demonstrated that gait contains relevant information regarding the degree of recovery [9–11]. Furthermore, Punt et al. demonstrated that gait is associated with the probability of falling in people after stroke [12]. However, it is yet unclear if and how gait after stroke is associated with their ability to walk in daily life. Therefore, to get a better understanding of gait recovery after stroke and specify and tailor interventions during rehabilitation, the additive value of measuring gait after stroke should be further explored.

In previous work, we demonstrated that gait after stroke in clinical rehabilitation can objectively and reliably be measured using IMUs [13]. In this study, 106 gait features in various domains were found to be reliable, indicating that a large quantity of information can be obtained from a single 2MWT. However, there are several drawbacks to the fact that so many gait features can be collected. First, it is likely that there is overlap in the information that these features contain, since the calculations used to determine the outcomes were similar (e.g., the average time per stride measured from the left and right foot sensor). Second, the large number of features and the complexity of the features makes it difficult for clinicians to interpret the outcomes. Lastly, it remains unclear what the added value of measuring gait with IMUs is, with respect to clinically relevant outcome measures. Accordingly, to facilitate the implementation of measuring gait using IMUs by clinicians, the dimensionality of outcomes should be reduced and, more importantly, the relevance of these outcomes must be determined.

An approach to explore the relevance of measuring gait with IMUs over gait speed is to identify the relationship between IMU-based gait features and the walking ability in daily life. There are several questionnaires and tests that are used to assess walking ability in people after stroke [3, 14]. A major disadvantage of these questionnaires and tests is that the outcomes are often subjective or measured in a lab setting. As an alternative, IMUs can objectively measure walking ability in daily life via gait features, such as the average gait speed and the number of steps per day [15, 16]. It is yet unclear if and how the information obtained from an IMU-instrumented 2MWT is associated with these measures of walking ability in addition to gait speed.

The aim of this study was to explore if gait features, measured with IMUs, improve the estimation of walking ability in daily life in people after stroke. This was done by assessing if IMU-based gait features significantly affect the relationship between gait speed and measures of walking ability.

## Methods

### Participants and study design

Longitudinal data from people after stroke in clinical stroke rehabilitation were collected. Participants were recruited in five clinical rehabilitation-centers in the Netherlands between January 1, 2021 and January 1, 2023. All participants were diagnosed with stroke according to the definition of the World Health Organization [17]. Inclusion criteria were 1) above the age of 18; 2) in the sub-acute or chronic phase after stroke; 3) signed the informed consent; 4) capable of understanding and performing simple tasks; 5) a Functional Ambulation Categories of at least 3. Participants were excluded if they were unable to walk at least 0.05 meters per second for two minutes [13]. Participants provided written informed consent prior to participating. This study was approved by the medical ethical review committee of Utrecht (METC number: 20-462/C). This study is reported following the STROBE guidelines [18].

### Procedure

At a three-week interval, an assessment was administered by a physiotherapist or trained research assistant during stroke rehabilitation, spanning from admission to discharge. The assessment consisted of two parts.

In the first part, during a clinical assessment, participants walked for two-minutes at self-selected speed on a fourteen-meter walking path with cones at both ends. Data were collected with three IMUs (manufactured by Aemics b.v. Oldenzaal, The Netherlands), located at the left and right foot and low back. The IMUs consisted of a triaxial accelerometer and gyroscope and measured with a sampling rate of 104 samples per second. The ranges of the accelerometer and gyroscope were set to 8m/s^2^ and 500°/s respectively. Participants were allowed to walk with a walking aid in the 2MWT. If the participant walked both with and without walking aid in daily life, the walking test was administered under both conditions [13]. In addition to the gait assessment, demographics (age, gender) and stroke-specific characteristics (stroke type and side) were collected and the following standard clinical tests were administered: Berg Balance Scale [19], Trunk Control Test [20], Motricity index [21], Modified ranking scale at admission [22], Barthel Index at admission [23] and the Functional Ambulation Categories both with and without walking aid [24].

In the second part of the assessment, participants were measured for two consecutive days, following the clinical assessment, with a single IMU. The same sensor was used as in the 2MWT, however only the sensor was placed at the calf, and only acceleration was measured with a sampling rate of 52 samples per second [25]. The sampling rate and location of the sensor were adjusted for measurement during daily life to enhance the battery life of the sensor and minimize the risk of sensor loss during the assessment.

### Data processing

After the assessment, the collected IMU-based gait and physical-activity measurements were uploaded in an online environment in which they were processed and stored.

The 2MWT data underwent resampling to 100 Hz and were adjusted for the gyroscope offset. Subsequently, 106 gait features, including spatio-temporal, frequency, complexity, and asymmetry features, were calculated per measurement. These features were utilized to characterize gait of people after stroke. A complete list of all the gait features is provided in Table A2.

The data of the IMU-measurement in daily life were split up into parts of 10 seconds before applying a previously trained convolutional neural network with long-term short-term memory to identify gait in daily life. The model was trained on a balanced dataset containing walking at gait speeds between 0.5 and 5 km/h, among other activities, such as sitting, lying, standing, and standing kitchen work. The model achieved an accuracy of 0.93 indicating an excellent ability to identify gait. Next, a step-detection algorithm was applied to count the total number of steps relative to the total wearing time, with a minimum of 8 hours. Finally, a sensor-fusion algorithm was applied to combine the accelerometer and gyroscope data and compute linear acceleration [26]. Next, a Zero Velocity Potential Update was applied to determine the stand- and swing phases during walking [27]. The linear acceleration in anterior-posterior direction during the swing phases was integrated twice to determine the position, which allowed us to calculate the total covered distance per epoch, and thus the gait speed. This allowed the calculation of the average gait speed and maximum gait speed per day. These three described measures were used to indicate walking ability.

### Statistical analysis

The statistical analysis consisted of three steps: 1) feature selection; 2) dimensionality reduction; and 3) linear mixed models. The feature selection and dimensionality reduction steps were applied to extract relevant information from the raw IMU data. These two steps resulted in a few gait features, which were then used to assess the additive value of the IMU measurement in the estimation of the measures of walking ability in daily life. All analyses were performed using Python (version 3.7.3). The used algorithms are available via: ‘https://github.com/RichardFel/PCA_gait’. The mathematical equations used in this study are described in in Felius et al. [13].

### Feature selection

First, all 106 gait features that demonstrated good to excellent test-retest reliability (ICC *≥* 0.75) in Felius et al. were calculated for all 2MWTs [13]. Second, a correlation matrix was created to compute the correlation coefficients between the gait features. If the correlation coefficient between two features was *>* 0.95, these features were considered identical. Subsequently, the feature with the highest summed overall correlation was excluded. Third, the Kaiser-Meyer-Olkin measure (KMO) was used as a measure of sampling adequacy and calculated for all features and per feature. An overall KMO and a KMO per feature of *>*0.7 and *>* 0*.*5 were considered acceptable for analysis [28]. Finally, the gait features were standardized by calculating z-scores.

### Dimensionality reduction

A Principal Component Analysis (PCA) was applied to reduce the amount of overlapping information in the 2MWT gait features. PCA has been applied to gait features in several studies [29–31]. It tries to explain the maximum amount of total variance by transforming the original variables into fewer linear principal components (PCs), while retaining as much information as possible [28]. In this study, a PCA was conducted on the z-scored gait features. The Kaiser’s criteria, which states that only PCs with an eigenvalue *>*1 should be considered, was used to determine which PCs should be retained [28]. The retained PCs were named based on the gait feature with the highest correlation to the PC.

To estimate the robustness and reliability of the PCs, the PCs were externally validated with previously collected data [13]. This data consists of test-retest measurements of gait in people after stroke, measured with a one-day interval. The same protocol was used as described in this study. First, gait features were calculated per measurement and transformed into z-scores. Next, the loadings of the PCA, computed with the longitudinal data, were used to transform the gait features of the test-retest data into PCs. The test-retest differences between the PCs were calculated using the root-mean-square error (RMSE) to estimate the generalizability. Additionally, the intraclass correlation coefficient (ICC 2.1) and its 95% confidence interval, the standard error of measurement (SEM) and the minimal detectable change (MDC) for the between-day reliability were calculated per component. An ICC of 0.5-0.75 was seen as moderate reliability, 0.75-0.9 as good, and >0.9 as excellent [32, 33].

### Linear mixed models

The relationship between gait speed, the PCs and the measures of walking ability was evaluated in three steps using Linear Mixed Models [34]. Linear Mixed Models, i.e., multilevel models, are an extension of standard linear models, containing both fixed and random effects. The random effects are added on a subject level, accounting for the correlation of repeated observations within subjects. This allows us to analyze the relationship between gait speed, the PCs and walking ability using longitudinal data.

First, the associations between the PCs, gait speed, and the measures of walking ability were estimated with the Pearson’s correlation coefficient. If the absolute correlation coefficient between two PCs, or a PC and gait speed was higher than *≥* 0.9, only one PC or gait speed was included in a linear model to prevent collinearity.

Second, a Linear Mixed Model was created per measure of walking ability to determine the association with gait speed obtained from the 2MWT. The participants were added as a random effect to the models. The PCs were added via a forward selection procedure, testing for confounding and effect modification. A confounder was marked as a percentual change of 10% of the gait speed coefficient, and effect modification was seen as significant if the interaction term had a *p*-value < 0.05 [34]. The forward selection procedure resulted in a definitive model per measure of walking ability. These definitive models contained gait speed and all PCs that were marked as confounders or effect modifiers. Last, the definitive models were compared with the gait speed only model via the normalised RMSE, Akaike information criterion (AIC), and Bayesian information criterion (BIC) [35, 36].

## Results

### Demographics and characteristics

**Fig. 1:**
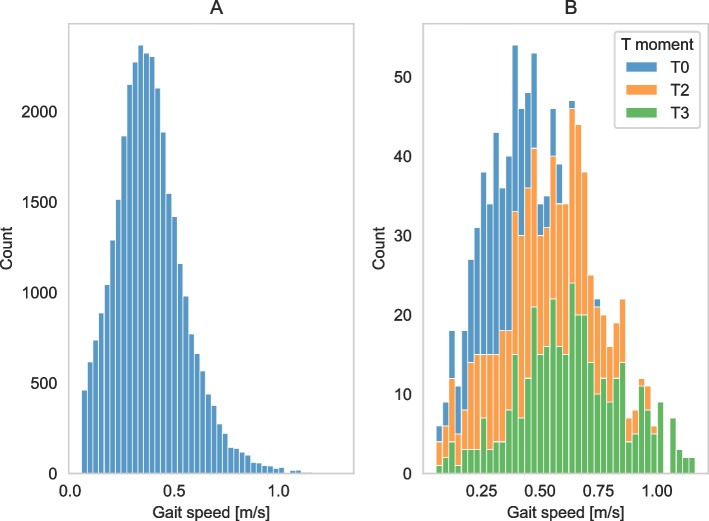
Distributions of gait speed in daily life. This figure illustrates the distribution of gait speed of all measurements of all participants (**A**), and a stacked distribution of three measurements of one randomly selected participant (**B**). In figure 1B, it is visible that the distribution of gait speed shifts to the right over time, indicating that the average and maximal gait speed increased over time. Gait speed measured with the corresponding 2MWT was 0.75m/s at T0, 1.16m/s at T2, and 1.28m/s at T3. Gait epochs with gait speed *≤*0.05 m/s were excluded from the analysis, as gait characteristics below this speed could not be determined reliably

Longitudinal data were collected from seventy-seven people after stroke during rehabilitation. The data consisted of 198 2MWT measurements and 135 corresponding measurements of walking ability. Participant and measurement characteristics are described in Table 1. Figure 1 visualizes the distribution of gait speed in daily life of all participants and that of one specific participant.

**Table 1:** Participant and measurement characteristics

**Patient Characteristics (** ***N*** ** = 77)**	Outcome
Gender	
Male	44
Female	33
Age in years	71.5 ± 12.8
Stroke type	
Ischemic	31
Haemorrhagic	24
Unknown	22
Stroke side	
Left	31
Right	36
Unknown	10
Barthel Index at admission (*N* = 73)	14.0 ± 4.6
Berg Balance Scale at admission (*N* = 72)	38.7 ± 15.2
FAC at admission	
With walking aid (*N* = 74)	2.6 ± 1.8
Without walking aid (*N* = 73)	2.6 ± 1.8
Trunk control test at admission (*N* = 70)	94.1 ± 14.9
Modified ranking scale at admission (*N* =54)	3.3 ± 0.8
**2MWT (** ***N*** ** = 198)**^**a**^	
Walking aid	
Yes	111
No	87
Gait speed (m/s)	0.71 ± 0.29
Mean stride time [s]	1.3 ± 0.4
Mean stride length [m]	0.9 ± 0.2
**Measures of walking ability in daily life (** ***N*** ** = 135)**	
Average gait speed [m/s]	0.37 ± 0.08
Maximum gait speed [m/s]	0.64 ± 0.15
Number of strides	2338 ± 1683

*Abbreviations*: *m* meter, *s* second, *FAC* Functional ambulation categories

^a^A selection of three features was made to give an impression of the outcomes of the 2MWT

### Feature selection

Thirty-six of the 106 gait features were removed due to a correlation coefficient *>* 0.95. No variables were excluded because of the Kaiser-Meyer-Olkin measure (KMO). The average overall KMO was 0.89, which is considered sufficient for the analysis. The PCA resulted in nine principal components (PC) with an eigenvalue of *>*1, accounting for 85.1% explained variance. The first PC accounted for 56.5% of the total explained variance. The other eight PCs accounted for 8.2%, 5.3%, 4.8%, 2.9%, 2.3%, 2.1%, 1.6%, 1.5% respectively. Based on the features with the highest correlation, the PCs were labelled: tempo, asymmetry, postural stability, trunk movement, stride variation, rhythm, stride intensity, stride distance, and stride regularity. The correlation between the PCs and the measures of walking ability is described in Table A1 in the Appendix. All PCs demonstrated an ICC-value of *>* 0*.*75 indicating a good to excellent reliability. The ICC and RMSE are described in Table A2 in the Appendix.

### Linear mixed models

#### Correlation coefficients

**Fig. 2:**
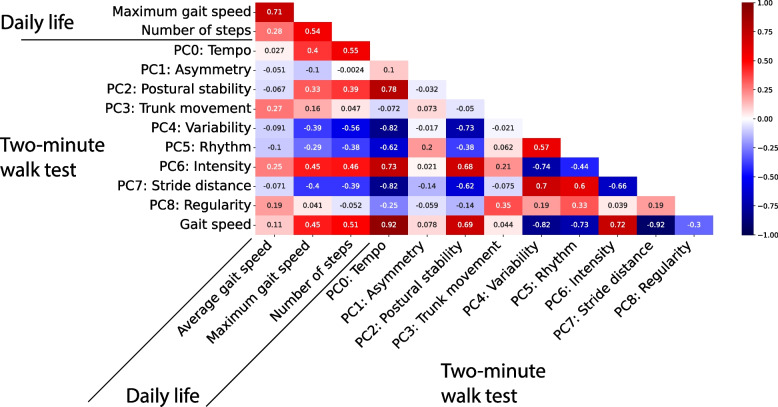
Correlation between the Principal components. Heatmap containing the Pearson’s correlation coefficients between measures of walking ability in daily life, the principal components (PCs) and the gait speed assessed using the 2MWT

The correlation coefficients between outcomes from the 2MWT and the measures of walking ability are visualized in Fig. 2. The gait speed obtained from the 2MWT was strongly correlated to tempo (PC0), postural stability (PC2), variability (PC4), rhythm (PC5), intensity (PC6), and stride distance (PC7). Moreover, the 2MWT gait speed was moderately correlated to the maximum gait speed and the number of steps in daily life. Asymmetry (PC1), Trunk movement (PC3), and Regularity (PC8) were weakly correlated to all other variables. To avoid collinearity, tempo (PC0) and stride distance (PC7) were not used in the same linear model as two-minute walk-test Gait speed.

#### Estimations

**Fig. 3:**
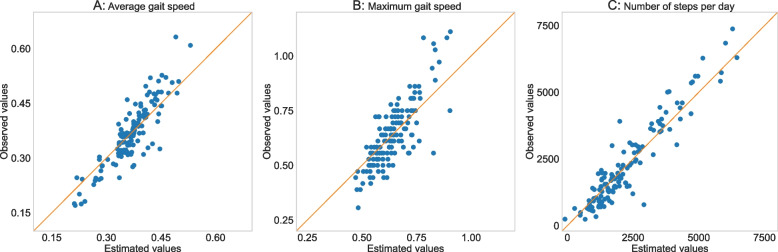
Difference between the observed and estimated values. Scatterplot of the estimated versus the observed value of the average gait speed (**A**), the maximum gait speed (**B**), and the number of steps (**C**) in daily life

The gait-speed-only Linear Mixed Models demonstrated a significant relationship between the 2MWT gait and the average gait speed, the maximal gait speed, and the number of steps. Only Intensity (PC6) was a confounder in the relationship for all three measures of walking ability. Additionally, an interaction effect was found between gait speed and Intensity (PC6) for the maximum gait speed and the number of steps. The nRMSE, AIC and BIC all indicated a comparable outcome of the gait speed only and the combined model (Table 2). The residuals of the definitive models were normally distributed, and the variance was homogeneous (Figure A1, A2). The overall estimation of the definitive models was weak for the average and maximum gait speed and moderate for the number of steps per day (Fig. 3).

**Table 2:** Description of the coefficients and model fit of the gait-speed-only model and the definitive model

		**Gait-speed-only model**			**Final model**		
	**Fixed**	**Coeff**	**SE**	**CI [95%]**	**Coeff**	**SE**	**CI [95%]**
Average gait speed [m/s]	Gait speed 2MWT [m/s]	0.067	0.031	[0.006,0.128]	-0.021	0.117	[-0.250,0.207]
	Intensity (PC6)	-	-	-	0.106	0.042	[0.025,0.188]
		**NRMSE**	**AIC**	**BIC**	**NRMSE**	**AIC**	**BIC**
		0.507	291	302	0.501	287	301
	**Fixed**	**Coeff**	**SE**	**CI [95%]**	**Coeff**	**SE**	**CI [95%]**
Max gait speed [m/s]	Gait speed 2MWT [m/s]	0.280	0.05	[0.189,0.379]	0.175	0.068	[0.040,0.306]
	Intensity (PC6)	-	-	-	0.088	0.071	[-0.05,0.227]
	Gait speed 2MWT *PC6				0.034	0.015	[0.004,0.064]
		**NRMSE**	**AIC**	**BIC**	**NRMSE**	**AIC**	**BIC**
		0.641	449	461	0.640	445	463
	**Fixed**	**Coeff**	**SE**	**CI [95%]**	**Coeff**	**SE**	**CI [95%]**
Number of steps	Gait speed 2MWT [m/s]	288	48	[193,383]	281	67	[150,412]
	Intensity (PC6)	-	-	-	-40	65	[-167,-87]
	Gait speed 2MWT *PC6				30	14	[4,57]
		**nRMSE**	**AIC**	**BIC**	**nRMSE**	**AIC**	**BIC**
		0.347	2253	2264	0.343	2251	2269

*Abbreviations*: *Coeff* Coefficient, *SE* Standard error, *CI* Confidence interval, *nRMSE* normalised root mean square error, *AIC* Akaike information criterion, *BIC* Bayesian information criterion, *m* meter, *s* seconds

The definitive model included gait speed and all PCs that were significant confounders or effect modifiers

## Discussion

We assessed the relevance of measuring gait in clinical stroke rehabilitation with Inertial Measurement Units (IMU), by evaluating the additive value of IMU-based outcome measures on the walking ability in people after stroke. We found that the estimation of measures of walking ability with a gait-speed-only linear mixed model resulted in a weak estimation of the average and maximum gait speed in daily life, and a moderate estimation of the number of steps per day. Moreover, we found that adding IMU-based gait features did not considerably improve these estimations. These findings suggest the existence of other factors, which may not be directly measurable from gait kinematics, that cause variability in the walking ability. Consequently, a simple measurement of gait speed, for instance with a 2MWT, might result in inaccurate estimations of someone’s ability to safely ambulate in daily life. Future research is essential to investigate factors, not directly measurable in gait, that are related to the ability to walk in daily life.

The principal component analysis, used to reduce the dimensionality of the gait features, resulted in nine principal components (PCs), with the first PC explaining 56.5% of the variance in the data. This high amount of explained variance in the first PC indicates that a large portion of the included gait features measured a similar construct, namely gait speed. Additionally, five of the eight remaining PCs were also strongly correlated to gait speed (Fig. 2). The prominent presence of gait speed in the data was to be expected since many of the included gait features measured a construct in the spatio-temporal domain and were highly correlated (Table A1). Moreover, the study of Huijben et al. demonstrated that gait speed affects many gait features, which strengthens this finding [37]. The first component being strongly correlated to gait speed is in line with the study of Olney et al., Morris et al. and Arcolin et al. [29–31]. A major difference with these studies is that we found more PCs, which might be due to a higher number of included gait features. For example, in the study of Arcolin et al. (2019) only eight gait features were included in the principal component analysis.

To identify whether the IMU-based gait features contain new relevant information, the PCs were added to the linear mixed models with the number of steps, average gait speed, and maximal gait speed as dependent variables. Our hypothesis was that new objective information about gait, such as asymmetry and variability, would improve the estimation the walking ability in daily life. The goodness of fit measures indicated that the model did not considerably improve with the added PCs. A possible explanation for the limited improvement of the models is that gait speed in itself is already determined by (and determines) several of the gait features that we assessed (as also shown by the high correlation between gait speed and the PC’s) [37–41]. This would suggest that a model including only the PC’s, would perform similar to a model containing only gait speed, which was confirmed in post-hoc analysis. Nevertheless, it remains unclear if gait after stroke influences the recovery trajectory in the long-term. Moreover, measuring gait might be used to personalize rehabilitation, since it allows monitoring of gait features, such as asymmetry, which in turn can be used to set accurate rehabilitation goals and tailor interventions. Overall, the estimations of measures walking ability in daily life of the definitive models were weak to moderate, including the gait-speed-only models. The absence of an association between gait speed and walking ability in daily life was unexpected, as previous studies reported an evident link between gait speed and walking ability [4–7]. Notably, prior studies categorized walking ability, while our approach involved continuous outcome measures. As a result, we made the assumption of a linear relationship between gait features and walking ability across all levels of gait, which may not hold true for all people after stroke. Moreover, a possible explanation for the weak-moderate estimation in general is that the dependent variables were an assessment of their performance, whereas the input can be considered a measurement of their capacity. These constructs are not necessarily correlated since a strong behavioral element is present in daily life [42]. Therefore, one could question the ecological validity of the 2MWT if it is used to estimate someone’s ability to walk in daily life [43].

In our study, the evaluation was limited to gait-related information for estimating daily life walking ability. However, it's conceivable that patient characteristics, such as age, balance, cognitive function, and fear of falling, also influence an individual’s walking ability in their daily routine [44–46]. Therefore, future research could benefit from incorporating a more diverse range of variables to enhance the accuracy of model estimations.

The methods used in this study have several limitations. First, the principal component analysis and the linear mixed models are both linear models, thereby assuming that the data can be modelled with a linear function. With the number of gait features that we included in the PCA, it is likely that this does not hold true for all features. As an alternative, non-linear techniques, such as a kernel-principal component analysis or a self-organizing map, could be explored to analyze and reduce the dimensionality of the data, without the assumption of linearity. The disadvantage of these techniques is that the results are often more difficult to interpret. Second, the principal component analysis resulted in nine PCs with an eigenvalue of greater than one. Based on previous studies, we expected to find fewer PCs, which would make it easier to label and interpret the outcomes [29–31]. Third, we used the PCA to reduce the amount of overlapping information from an IMU-based gait measurement and maintained a limited number of principal components. Specifically, nine principal components were maintained and these accounted for a large percentage (85%) of the variance in our data. However, the magnitude of the explained variance is not necessarily related to the clinical relevance. For example, it is theoretically possible that a relatively small principal component contains information that is clinically relevant. Nevertheless, including more variables in hypothesis testing increases the probability of finding false positives. Finally, a relatively small number of participants was used to compute the linear mixed model. As a result, the goodness of fit measures might lack precision. In future work, a larger sample size is recommended.

Our overarching goal is to develop an instrumented test that clinicians can use to monitor individual progression during stroke rehabilitation. Therefore, to increase the interpretability of the outcomes, we reduced the dimensionality of the data with more than 90% while maintaining 85% of the variance. The resulting PCs might be easier to use in clinical practice to evaluate and monitor gait in comparison to the raw gait features. However, additional research is required to demonstrate the clinical relevance of measuring gait with IMUs compared to conventional testing methods. Further work is in progress to indicate if the PCs are responsive to gait rehabilitation and can thus be used to monitor progression. Additionally, further work should indicate if the PCs have added predictive value with respect to other relevant outcomes during recovery, such as fall risk during and after rehabilitation, and gait independence at six months after stroke.

## Conclusions

We evaluated the relevance of measuring gait after stroke in addition to gait speed for the estimation of their walking ability in daily life. We found that gait speed measured with a 2MWT in people in clinical stroke rehabilitation results in a weak to moderate estimation of the walking ability in daily life. Measuring gait after stroke using inertial measurement units does not improve this estimation. Therefore, estimating the walking ability in daily life using only gait features measured in clinical practice, might be inaccurate. Future research is needed to explore these discrepancies across various walking tests and settings to better understand the differences between clinical gait assessments and walking ability in daily life. Moreover, future research should identify non-gait factors that influence the walking ability in daily life.

### Supplementary Information

**Supplementary Material 1.**

## Data Availability

Data will be made available on request in 2024, after the research project ‘making sense of sensor data for personalized healthcare’ is finished.

## Abbreviations

IMU: Inertial Measurement Unit
PC: Principal Component
PCA: Principal Component Analysis
2MWT: Two-minute Walk Test
FAC: Functional Ambulation Categories
ICC: Intraclass Correlation Coefficient
KMO: Kaiser-Meyer-Olkin measure
SEM: Standard Error of <measurement
MDC: Minimal Detectable Change
RMSE: Root Mean Square Error
AIC: Akaike Information Criterion
BIC: Bayesian Information Criterion
