# Supplementary material for: Beyond gait speed: exploring the added value of Inertial Measurement Unit-based measurements of gait in the estimation of the walking ability in daily life
Source: BMC Neurol. 2024 Apr 17;24:129. doi: 10.1186/s12883-024-03632-0 (PMC11022465; doi:10.1186/s12883-024-03632-0)
Supplement: Supplementary file 1 — Supplementary Material 1. [file 12883_2024_3632_MOESM1_ESM.docx]

# Appendix

# Table A1: Absolute correlation coefficient of the principal components and the gait features

|  | PCA_0 | PCA_1 | PCA_2 | PCA_3 | PCA_4 | PCA_5 | PCA_6 | PCA_7 | PCA_8 |
| --- | --- | --- | --- | --- | --- | --- | --- | --- | --- |
| ACOR acc AP R | **0.95** |  | **0.77** |  | **0.83** | 0.61 | **0.74** | **0.79** |  |
| ACOV gyr MR R | **0.93** |  | **0.71** |  | **0.79** | 0.62 | **0.73** | **0.83** |  |
| ACOV acc VT L | **0.93** |  | **0.79** |  | **0.84** | 0.58 | **0.75** | **0.76** |  |
| ACOV acc VT R | **0.93** |  | **0.74** |  | **0.76** | 0.57 | **0.72** | **0.79** |  |
| RMS acc VT R | **0.92** |  | **0.71** |  | **0.81** | 0.66 | **0.82** | **0.85** |  |
| KMPH R | **0.92** |  | 0.69 |  | **0.82** | **0.73** | **0.72** | **0.92** | 0.30 |
| ACOV acc AP L | **0.91** |  | **0.79** |  | **0.89** | 0.56 | **0.73** | **0.70** |  |
| ACOV gyr ML L | **0.91** |  | 0.66 |  | **0.77** | 0.65 | 0.63 | **0.82** | 0.35 |
| RMS acc VT L | **0.91** |  | **0.77** |  | **0.88** | 0.64 | **0.88** | **0.78** |  |
| Stride vel mean L | **0.90** |  | 0.68 |  | **0.80** | 0.66 | 0.66 | **0.83** | 0.38 |
| RMS gyr VT L | **0.88** |  | **0.73** |  | **0.86** | 0.54 | **0.79** | **0.79** |  |
| Stride vel mean R | **0.87** |  | 0.66 |  | **0.71** | 0.56 | 0.64 | **0.87** |  |
| ACOV gyr AP B | **0.87** |  | **0.81** |  | **0.71** | 0.42 | 0.67 | **0.77** |  |
| ACOV acc MR R | **0.86** |  | **0.79** |  | **0.92** | 0.53 | 0.70 | 0.66 |  |
| ACOV gyr AP R | **0.86** |  | 0.68 |  | **0.81** | 0.55 | 0.58 | **0.77** | 0.34 |
| Rms acc AP B | **0.86** |  | **0.88** |  | **0.82** | 0.46 | **0.84** | **0.76** |  |
| SI standphases | **0.86** |  | 0.66 |  | **0.86** | **0.77** | 0.69 | **0.76** |  |
| Range gyr VT B | **0.85** |  | **0.82** |  | **0.73** | 0.39 | **0.73** | **0.79** |  |
| ACOV acc AP B | **0.84** |  | **0.88** |  | **0.88** | 0.47 | **0.73** | 0.68 |  |
| ACOV acc VT B | **0.83** |  | **0.89** |  | **0.74** | 0.44 | **0.70** | **0.72** |  |
| Cadence L | **0.83** |  | 0.56 |  | **0.77** | **0.85** | 0.61 | **0.73** | 0.32 |
| RMS gyr ML L | **0.82** |  | 0.68 |  | 0.68 | 0.58 | 0.56 | **0.72** |  |
| RMS gyr MR R | **0.82** |  | 0.56 |  | 0.47 | 0.53 | 0.54 | **0.71** |  |
| RMS gyr VT R | **0.82** |  | 0.61 |  | **0.78** | 0.57 | **0.72** | **0.85** |  |
| Stride vel std R | **0.81** |  | 0.65 |  | 0.66 | 0.51 | 0.62 | **0.89** |  |
| Range gyr VT L | **0.81** |  | 0.66 |  | **0.80** | 0.43 | **0.77** | **0.75** |  |
| Rms gyr AP B | **0.80** |  | **0.93** |  | 0.68 | 0.44 | 0.64 | 0.68 |  |
| Range acc VT B | **0.80** |  | **0.84** |  | **0.81** | 0.47 | **0.85** | 0.65 |  |
| Stride vel std L | **0.79** |  | 0.70 |  | 0.64 | 0.47 | 0.66 | **0.87** |  |
| Range acc AP B | **0.78** |  | **0.81** |  | **0.80** | 0.42 | **0.88** | **0.71** |  |
| Rms acc VT B | **0.78** |  | **0.86** |  | **0.75** | 0.38 | **0.74** | 0.64 |  |
| ACOV gyr AP L | **0.78** |  | **0.71** |  | **0.88** | 0.49 | 0.64 | 0.61 |  |
| Range gyr AP L | **0.78** |  | 0.55 |  | **0.82** | 0.61 | **0.71** | **0.73** |  |
| ACOV acc ML L | **0.77** |  | **0.81** |  | **0.88** | 0.43 | **0.71** | 0.54 |  |
| ACOV acc ML B | **0.77** |  | **0.93** |  | 0.66 | 0.40 | 0.64 | 0.64 |  |
| Stride dist mean R | **0.77** |  | 0.60 |  | 0.64 | 0.55 | 0.62 | **0.89** |  |
| ACOV gyr VT B | **0.76** |  | **0.88** |  | 0.61 | 0.41 | 0.52 | 0.65 |  |
| Range acc AP L | **0.75** |  | 0.62 |  | 0.68 | 0.51 | **0.84** | **0.71** |  |
| RMS acc ML L | **0.75** |  | 0.53 |  | **0.80** | 0.63 | 0.69 | 0.67 |  |
| ACOV gyr VT L | **0.72** |  | **0.72** |  | **0.88** | 0.39 | **0.72** | 0.51 |  |
| Range gyr VT R | **0.71** |  | 0.56 |  | **0.73** | 0.40 | **0.71** | **0.78** |  |
| Range gyr AP R | 0.68 |  | 0.56 |  | **0.86** | 0.54 | 0.69 | **0.74** |  |
| Stride time mean R | 0.68 |  | 0.41 |  | 0.61 | **0.92** | 0.48 | 0.66 | 0.34 |
| Range acc ML B | 0.66 |  | **0.80** |  | 0.68 | 0.33 | **0.86** | 0.55 |  |
| Rms gyr ML B | 0.66 |  | **0.95** |  | 0.69 | 0.31 | 0.63 | 0.48 |  |
| Range gyr ML B | 0.65 |  | **0.90** |  | **0.74** | 0.32 | 0.70 | 0.49 |  |
| ACOV gyr VT R | 0.63 |  | 0.53 |  | **0.84** | 0.46 | 0.58 | 0.58 |  |
| SI swingphases | 0.62 | 0.66 | 0.39 |  | 0.51 | 0.48 | 0.44 | 0.48 |  |
| Range acc AP R | 0.61 |  | 0.43 |  | 0.36 | 0.37 | **0.72** | 0.64 |  |
| HR VT B | 0.59 |  |  | 0.32 | 0.51 | 0.56 |  | 0.66 | 0.34 |
| Range acc VT R | 0.59 |  | 0.40 |  | 0.50 | 0.36 | **0.79** | 0.58 |  |
| RMS gyr AP R | 0.58 |  | 0.48 |  | **0.84** | 0.55 | 0.60 | 0.64 |  |
| Range acc MR R | 0.55 |  | 0.44 |  | 0.67 | 0.40 | **0.80** | 0.64 |  |
| steps_per_day_y | 0.55 |  | 0.39 |  | 0.56 | 0.38 | 0.46 | 0.39 |  |
| ACOV gyr ML B | 0.52 |  | **0.88** |  | 0.55 |  | 0.46 | 0.36 |  |
| Range acc VT L | 0.52 |  | 0.46 |  | 0.56 | 0.37 | **0.85** | 0.47 |  |
| Dominant peak density R | 0.47 |  | 0.33 | 0.41 | 0.37 | 0.44 |  | 0.37 | **0.88** |
| HR AP B | 0.45 |  |  |  | 0.41 | 0.53 |  | 0.58 |  |
| Stride time std L | 0.44 |  |  |  | 0.36 | **0.89** |  | 0.38 | 0.35 |
| Stride time norm L | 0.44 |  |  |  | 0.33 | **0.73** |  | 0.31 | 0.42 |
| HR ML B | 0.44 |  | 0.36 | 0.56 |  | 0.32 |  | 0.37 |  |
| Dominant peak slope R | 0.37 |  |  | 0.40 |  | 0.38 |  | 0.31 | **0.91** |
| Stride dist std R | 0.35 |  | 0.30 |  |  | 0.36 | 0.38 | **0.74** |  |
| Dominant peak density ML B |  |  |  | **0.92** |  |  |  |  | 0.31 |
| Stride dist std L |  |  |  |  |  |  |  | 0.40 |  |
| IH ML B |  |  |  | **0.83** |  |  | 0.36 | 0.38 |  |
| SI Swing/stand |  | 0.70 |  |  |  | **0.75** |  | 0.31 |  |
| SR Swing/stand |  | **0.76** |  |  |  | 0.65 |  |  |  |
| GA swingphases |  | **0.92** |  |  |  | 0.42 |  |  |  |
| GA Swing/stand |  | **0.89** |  |  |  | 0.38 |  |  |  |
| SR standphasess |  | **0.92** |  |  |  |  |  |  |  |
| The Pearson’s correlation coefficient is used to determine the correlation. Values greater than 0.7 are marked in bolt. Values below 0.3 were excluded from this table.  Abbreviations: L = Left foot; R = Right foot; B = low back; Gyr = Gyroscope; Acc = Acceleration; Dist = Distance; KMPH = kilometres per hour; Vel = Velocity; DF = Dominant Frequency; LDE = local divergence exponent; ApproxE = Approximate entropy; SampleE = Sample entropy; std = Standard deviation; rms = Root Mean Square; AP = Anterior--posterior; ML = Medio-lateral; VT = Vertical; ACOV = Autocovariance; ACOR = Autocorrelation; HR = Harmonic ratio; IH = Index of harmonicity; SR = Symmetry ratio; SI = Symmetry index; GA = Gait asymmetry; SA= Symmetry Angle; Amp = Amplitude; Norm = Normalised. | | | | | | | | | |

Table A2: Reliability

|  | ICC [-CI, CI] | MDC (SEM) | RMSE |
| --- | --- | --- | --- |
| PC0: Tempo | 0.972 [0.93,0.99] | 2.463 (0.889) | 1.281 |
| PC1: Asymmetry | 0.959 [0.91,0.98] | 2.416 (0.872) | 1.255 |
| PC2: Postural stability | 0.899 [0.79,0.95] | 2.359 (0.851) | 1.224 |
| PC3: Trunk movement | 0.869 [0.73,0.94] | 2.0 (0.722) | 1.042 |
| PC4: Variability | 0.83 [0.66,0.92] | 1.575 (0.568) | 0.817 |
| PC5: Rhythm | 0.908 [0.81,0.96] | 1.308 (0.472) | 0.679 |
| PC6: Intensity | 0.899 [0.79,0.95] | 1.128 (0.407) | 0.587 |
| PC7: Stride distance | 0.805 [0.61,0.91] | 1.454 (0.525) | 0.753 |
| PC8 Regularity | 0.862 [0.71,0.94] | 1.422 (0.513) | 0.741 |
| Test-retest results of the PC transformed test-retest data collected in the study of Felius et al (2022) [7]. An Interclass correlation coefficient (ICC) of >0.7 was seen as a good reliability, and an ICC of >0.9 as excellent reliability. | | | |


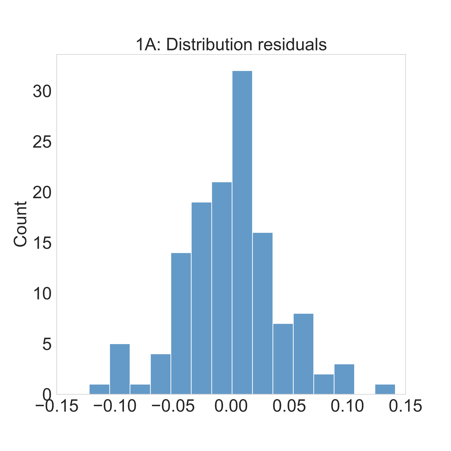

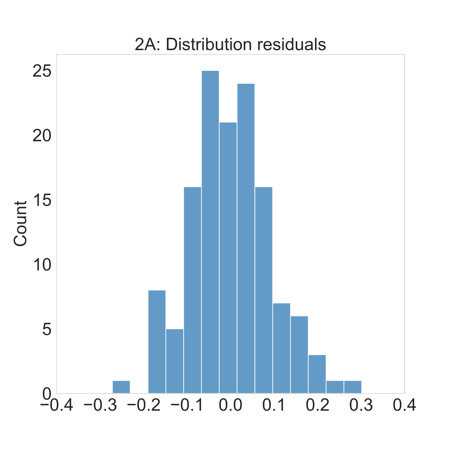

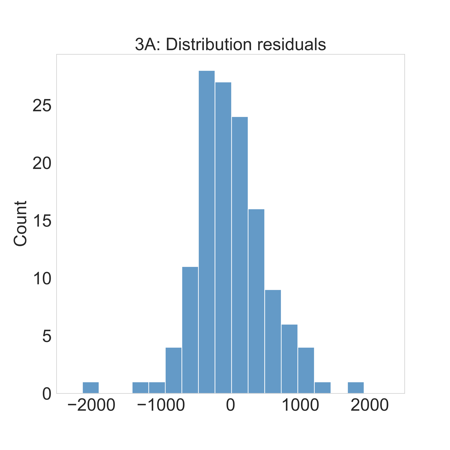
 Figure A1: Distributions of the residuals of the average gait speed (1A), maximal gait speed (2A) and number of steps per day (3A). A normal distributions is visible in all three plots, with a more leptokurtic distribution in the residuals of the steps per day (3A). From the Quantile-Quantile plot, i.e. a probability plot in which the distribution is compared to the standard, it is visible that the distribution of the residuals is Gaussian for average gait speed (1B), maximal gait speed (2B) and the number of steps per day (3B). Last, the residuals versus the fitted values for the average gait speed (1C), maximal gait speed (2C), and the number of steps per day (3C). These three plots indicate that the assumption of linearity is reasonable, the variance of the error terms is equal and there are no evident outliers.


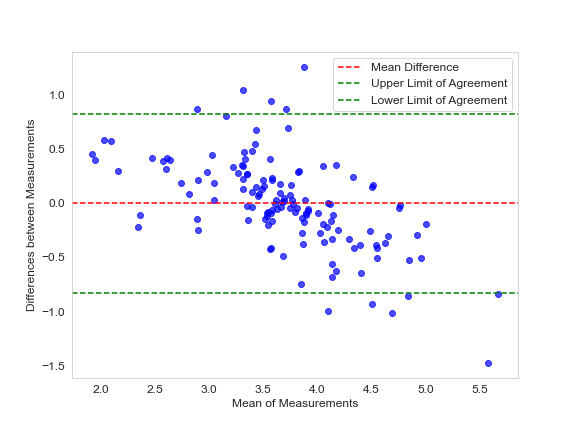

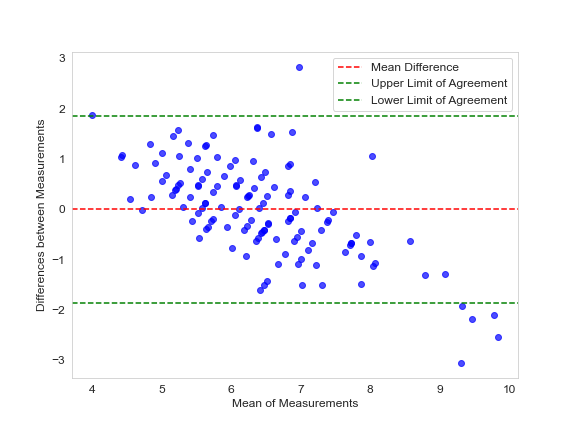

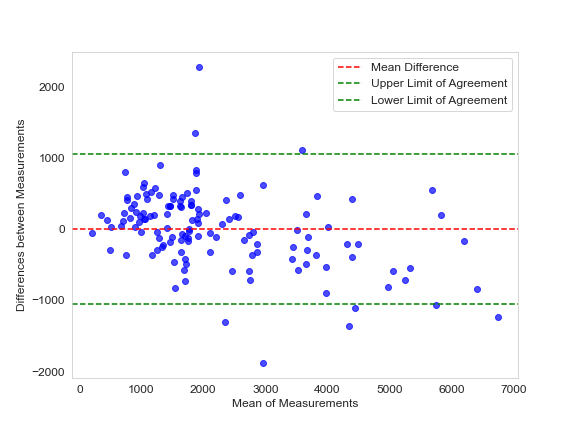


Figure A2: Bland-Altman plots of the observed and estimated average gait speed (left), maximal gait speed (middle) and the number of steps per day (right). The central line indicates a mean difference close to zero. The uniform spread of the points around the central line indicates homoscedasticity.
